# Supplementary material for: Narrative review after post-hoc trial analysis of factors that predict corneal endothelial cell loss after phacoemulsification: Tips for improving cataract surgery research
Source: PLoS One. 2024 Mar 21;19(3):e0298795. doi: 10.1371/journal.pone.0298795 (PMC10956851; doi:10.1371/journal.pone.0298795)
Supplement: S1 Fig — CD, cataract grade; EPT, effective phaco time. (DOCX) [file pone.0298795.s001.docx]

**Supplementary Fig S1. Plot of correlation coefficients in the literature.** CD, cataract grade; EPT, effective phaco time.
