# Supplementary material for: Narrative review after post-hoc trial analysis of factors that predict corneal endothelial cell loss after phacoemulsification: Tips for improving cataract surgery research
Source: PLoS One. 2024 Mar 21;19(3):e0298795. doi: 10.1371/journal.pone.0298795 (PMC10956851; doi:10.1371/journal.pone.0298795)
Supplement: S1 Table — (DOCX) [file pone.0298795.s002.docx]

## Supplementary Table S1.

Objective, number of eyes, technical details, eligibility criteria, and ECL reported by the studies shown in Table 1

| **Name country year** | **Type of study** | **No. eyes** | **Phacoemulsification method &**  **ECD measurement system** | **Elibibility criteria listed in paper (control group only** ^a^**)** | **ECL time**  **point** | **ECL** |
| --- | --- | --- | --- | --- | --- | --- |
| Our study  2022 | Ancillary analysis of RCT comparing DAC and a supracapsular phacoemulsification method | 275 | Stellaris (Bausch&Lomb), 2.2-mm incision, DuoVisc, DAC or Garde á vous, longitudinal, one experienced operator  Noncontact specular microscopy | Excl: Any cornea, ocular tone, or posterior segment pathology; history of retinal detachment, ocular trauma, or anterior/posterior segment surgery; additional procedures; BCVA <+0.2 logMAR; diabetes with insulin dependence/retinal complications; preoperative ECD <1500 cells/mm^2^; brown/white cataracts | 3 mo | 13% |
| Mahmoud Eqypt 2021 | Prospective study comparing patients with and without DM ^a^ | 32 with DM, 32 non-DM | Infiniti with Ozil (Alcon), 2.2-mm incision, methycellulose OVD, Stop&chop, torsional, one experienced operator  Noncontact specular imaging | Incl.: Senile nuclear cataract; LOCSIII NI and NII; normal fundus examination; normal IOP; 50–70 years.  Excl.: Diabetes, corneal pathology; low endothelial cell count (<2000 cells/mm2), poor pupillary dilatation; pseudoexfoliation syndrome; glaucomatous optic neuropathy; previous history of ocular trauma or uveitis; previous eye surgery; current or previous macular edema, maculopathy or retinopathy; cataract density interfered with preoperative OCT | 6 mo | 4.8% & 6.4%  NS |
| Budiman Indonesia 2021 | Prospective study | 148 | WhiteStar Signature PRO (Abbott Medical Optics), 2.75-mm incision, Healon5 twice, phaco-chop or stop-chop, 2 experienced surgeons  Noncontact specular microscopy | Incl.: cataract grade II-IV; age >30 years old, and under- went phacoemulsification with IOL implantation. Patients with preoperat;  Excl.: IOP >21 mmHg; ECD <1500/mm^2^; history of intraocular surgery, pseudoexfoliative syndrome; zonnular weakness; patients without IOL implantation; complication during or after surgery | 1 w  1 mo | 21% for ACD ≥3 mm vs 27% for ACD <3 mm p=0.03  28% for ACD ≥3 mm vs 36% for ACD <3 mm p=0.01 |
| Beato Portugal 2021 | Prospective study comparing ECL in DM and nondiabetic patients ^a^ | 45 DM, 43 non-DM | Infiniti (Alcon), 2.75-mm incision, Provisc, chop method not specified, longitudinal?, multiple experienced operators  Noncontact specular microscopy | Excl.: Prior eye surgery or trauma; any corneal, retinal, or optic nerve pathology; mature cataracts (brown/white); IOP >25 mmHg; pseudoexfoliation syndrome; current glucocorticoid treatment; ECD <1500 cells/m^2^; no diabetes | 1 mo  6 mo | 14.6% & 20.7%  15.7% & 20.7%  NS |
| Joo Korea 2021 | Retrospective study comparing ECL in DM and nondiabetic patients | 56 DM, 37 non-DM | Sovereign Compact (Abbott Medical Optics), 2.8-mm incision, OVD and chop method not specified, two experienced operators  Noncontact specular microscopy | Incl.: grade 2-3 LOCSIII cataract.  Excl.: history of ocular surgery, dry eye disease, uveitis or ocular inflammation, corneal opacities, glaucoma, and ocular trauma. | 12 mo | 13.3% & 11.4% |
| Dzhaber Germany 2020 | RCT comparing FLACS and pop-and-chop phacoemulsification ^a^ | 134, one eye FLACS, one eye phacoemulsification | Infiniti (Alcon), 2.75-mm incision, Provisc, Pop&Chop, longitudinal?, one experienced operator  Noncontact specular microscopy | Incl.: ≥18 years old; bilateral visually significant cataract; able to attend follow-up for at least 3 months  Excl.: FECD or other ocular pathology; previous/concurrent ocular surgery | 1 mo  3 mo | 10.7 & 6.8%  11.2% & 8.0%  Both NS |
| Choi Korea 2019 | Retrospective study looking at ECL >10 y after uncomplicated cataract surgery | 81 | Millenium (Bausch&Lomb), 2.75-mm incision, Healon, phaco-chop, longitudinal, one experienced operator  Noncontact specular microscopy | Incl.: uneventful phacoemulsification and posterior chamber IOL implantation  Excl.: Primary endotheliopathy (e.g. FECD or posterior polymorphous dystrophy); history of uveitis; zonulopathy (e.g. trauma); complicated cataract surgery (e.g. posterior capsule rupture); vitreous loss; other intraocular surgery before and after cataract surgery; laser iridotomy; conversion to extracapsular cataract extraction; IOL decentration; exacerbated postoperative inflammation (toxic anterior segment syndrome, uveitis) with ocular hypertension, infection; pseudoexfoliation; preoperative endothelial cell count <1900 cells/mm2; diabetes mellitus; preoperative small pupil (<6.0 mm) after mydriasis | >10 y | 20.6% |
| Krarup Denmark 2019 | RCT comparing FLACS and conventional phacoemulsification ^a^ | 96, one eye with FLACS, one with phacoemulsification | Infiniti (Alcon), 2.4-mm incision, Healon, DAC, torsional, one experienced operator  Noncontact specular microscopy | Incl.: visually significant cataract of any type and degree; age >18 years  Excl.: history of severe dry eye, herpetic keratitis, or uveitis; corneal scars; signs of keratoconus; pseudoexfoliation syndrome; uncontrolled glaucoma; visually significant maculopathy; vitreomacular traction; lack of cooperation or tremor; previous ocular surgery | 40 d  6 mo | 12.9% & 18%  13.6% & 17%  P=0.027 & 0.036 |
| Ganesan India 2019 | Case control study comparing diabetics and non-diabetics ^a^ | 80 in each group | Machine and incision size not indicated, viscodispersive, stop&chop, longitudinal, two experienced operators  Noncontact specular microscopy | Incl.: >40 years; nuclear sclerosis cataract  Excl.: High myopia (>-6D): corneal opacities; pseudoexfoliation; uveitis | 1 wk  6 wks  3 mo | 12.4%  16.6%  18.1% |
| Perone France 2018 | Prospective study on the correlation between CCT and ECL | 85 | Stellaris (Bausch&Lomb), 2.2-mm incision, OVD not specified, longitudinal, one experienced operator  Noncontact specular microscopy | Incl.: >18 years  Excl.: Total white cataract; history of ocular surgery; other eye diseases (corneal pathology, FECD, uveitis, glaucoma); systemic diseases (diabetic disorder and dementia) with the potential to affect vision | 2 h  4 d  15 d  1 mo | 3.0%  9.0%  10%  11% |
| Al-Osaily Saudi Arabia 2018 | Retrospective study on the corelations between ECL and cataract density | 71 | Whitestar Signature (Abbot Medical Optics), 2.2-mm incision, OVD not indicated, stop&shop, one experienced operator  Specular microscopy | Excl.: History of ocular surgery. | 1 mo | 17.0% |
| Singh 2017 India | RCT comparing MICS and phacoemulsification | 152: 27 MICS, 125 phacoemulsification | Infiniti (Alcon), 2.85-mm incision, 2% hydroxy propyl methy cellulose, Ringer’s lactate, other details unclear  Non-contact specular microscopy | Incl.: uncomplicated senile cataract; LOCSIII 3–4  Excl.: ECD <1500 cells/mm^2^; history of previous ocular surgery; coexisting ocular disease; intraop/postoperative surgical complications | 6 w | 7.3% & 13.7% |
| 6Misra NZ 2015 | Prospective study comparing patients with and without DM ^a^ | 28 DM, 23 non-DM | Stellaris (Bausch&Lomb), 2.85-mm incision, OVD and chopping technique not specified, longitudinal, two experienced operators | Excl.: contact lens wear; history of surgery or ocular trauma; complicated cataract surgery; sutures were required; concurrent nondiabetic corneal pathologies, pseudoexfoliation | 3 mo | 0.9% & 9%  P=0.007 |
| Mehra India 2015 | Prospective study examining the factors that affect ECL | 500 | Machine not specified; 2.2-mm incision; 3% sodium hyaluronate followed by dispersive OVD, phaco chop, longitudinal?, number of operators not indicated  Specular microscopy | Incl.: 40–70 years; nucleus sclerosis grades 1–4; ECD 1500–3000 cells/mm2 Excl.: diabetes mellitus; previous intraocular surgery or ocular trauma; oreoperative diagnosis of glaucoma; pseudoexfoliation or corneal endothelial dystrophy; complicated cataract surgery (iris trauma, Descemet’s detachment); posterior capsule rupture with vitreous loss | 6 w | 14.5% |
| Atas Turkey 2014 | Prospective case series comparing transversal and torsional phacoemulsification | 35 transversal, 51 torsional | WhiteStar Signature Ellips FX (Advanced Medical Optics) or Infiniti Ozil IP (Alcon), 2.8-mm incision, Viscoat, quick-chop, transversal or torsional, number of operators not indicated  Noncontact specular microscopy | Incl.: >50 years; senile nuclear cataract  Excl.: Coexisting ocular disease (ocular surface, corneal, or retinal diseases); history of trauma/surgery/inflammation; pseudoexfoliation; poor papillary dilatation; ECD <1500 cells/mm2; corneal dystrophies; complicated surgeries (e.g. posterior capsule tear and zonular dialysis) | 1 mo | 7% & 6%  NS |
| Mayer Germany 2014 | Retrospective case series comparing FLACS and manual phacoemulsification ^a^ | 88 FLACS, 62 phacoemulsification | Infiniti (Alcon), 2.2–2.4-mm incision, Provisc, DAC, longitudinal?, one experienced operator  Noncontact specular microscopy | Excl.: pseudoexfoliation syndrome; past glaucoma filtration surgery; optic atrophy; ocular tumors; prior vitrectomy; cataract/refractive lens surgery; central corneal scarring | 1 mo | 4.7% & 6.7%  p=0.02 |
| Orski Poland 2014 | Retrospective study on factors affecting ECL | 365: 68 with 1.8-mm incision, 297 with 2.75-mm incision | Machine not specified, 1.8 or 2.75-mm incision, OVD, stop&chop, four operators | None specified. | 1 mo | Total 13.1%  LOCS 1-2 6.4%  LOCS 3 10.6%  LOCS 4 12.0%  LOCS 5 18.1%  LOCS 6 31.6% |
| Conrad  Germany 2013 | RCT comparing FLACS and conventional phacoemulsification ^a^ | 73, one FLACS, one phacoemulsification | Stellaris (Bausch&Lomb), 2.75-mm incision, healon, Stop&Chop, longitudinal, one experienced operator  Noncontact specular microscopy | Incl.: visually significant cataract; dilated pupil width of >6.0 mm  Excl.: history of serious coexisting ocular disease; uncontrolled glaucoma; optic atrophy; ocular tumors; use of topical or systemic steroids or NSAIDs during the previous 3 months; relevant corneal opacities; known zonular weakness; age <22 years | 1 wk  6 wks  3 mo | 7.9% & 12.1%  8.0% & 11.1%  8.1% & 13.7%  p<0.001 |
| Soliman Egypt 2012 | Prospective case series examining influence of microcoaxial phacoemulsification parameters | 120 | Infiniti, 2.2-mm incision, Viscoat, Stop&Chop, torsional, number of operators not indicated  Noncontact specular microscopy | Incl: LOCSIII grade I–V nuclear or corticonuclear cataract  Excl.: Cataract hardness >NO5; coexisting ocular disease; pseudoexfoliation; poor pupillary dilatation; corneal dystrophy; corneal scarring; ECD <1500 cells/mm2; age-related macular degeneration; glaucoma; history of trauma or surgery | 3 mo | 15.4% |
| Gonen Turkey 2012 | RCT comparing biaxial torsional or biaxial longitudinal phacoemulsification in high grade cataract | 70 | Infiniti (Alcon), 2.8-mm incision, Microvisc, Quick-chop, longitudinal or torsional, one experienced operator  Noncontact specular microscopy | Incl.: >50 years; age-related LOCSIII grade 4–5 nuclear cataract; ECD >1500 cells/mm2  Excl.: Corneal pathology (e.g. stromal or endothelial dystrophy); zonular weakness; pseudoexfoliation; dilated pupil size <7.0 mm; high myopia; diabetic retinopathy or senile macular degeneration; history of intraocular surgery, glaucoma, or uveitis | 1 mo  3 mo | 39.1% & 36.5%  38.6% & 35.4%  NS |
| Takacs Hungary 2012 | RCT comparing FLACS and CPS | 38 in each group |  | Excl. Patients showing low cooperation; dense (grade ≥4) or white cataract corneal scars or opacities; anterior segment abnormalities; floppy iris syndrome; poor pupillary dilation | 1 mo | 4% & 11% |
| Faramarzi Iran 2011 | RCT comparing bevel-up and bevel-down phacoemulsification | 60 eyes (30 & 30) | WhiteStar Sovereign (Advanced Medical Optics), 2.8-mm incision, OVD not mentioned, Stop&Chop, longitudinal, one experienced operator  Noncontact specular microscopy | Incl.: Moderate lens opacity  Excl.: History of significant ocular trauma or intraocular surgery; corneal pathology; pseudoexfoliation syndrome; intraocular inflammation; preoperative ECD <1800 cells/mm2; preoperative fully dilated pupil <6.0 mm; glaucoma; diabetes mellitus; surgical complications (anterior or posterior capsule tear); postoperative inflammation | 3 mo | 5.9% & 13.6%  P=0.012 |
| Mathew India 2011 | Case-control study comparing age matched patients with and without diabetes ^a^ | 158 DM, 165 non-DM | Manual SICS, machine, incision length, OVD, and chopping technique not detailed, multiple experienced operators  Noncontact specular microscopy | Incl.: 40–70 years  Excl.: Preexisting corneal diseases; glaucoma; uveitis; prior laser treatment | 6 w  3 mo | 9.3% & 7.7%  19.2% & 16.6%  P<0.05 |
| Lucena Brazil 2011 | RCT comparing BSS Plus and Ringers lactate | 50 BSS, 50 Ringers Lactate | Infiniti (Alcon), 2.75-mm incision, Vistagel, phaco-chop, longitudinal?, one experienced operator  Noncontact specular microscopy | Excl.: Not age-related cataract (e.g. secondary or congenital); previous ocular surgery or corneal disease; anterior chamber cells or flare; any condition that impeded corneal evaluation by specular microscopy and pachymetry or follow-up | 1 d  1 wk  2 wks  1 mo  2 mo | 12.7% & 10.3%  12.1% & 12.1%  11.3% & 13.5%  11.7% & 13.0%  9.2% & 13.1%  NS |
| Reuschel Germany 2010 | RCT comparing torsional and longitudinal mode phacoemulsification | 182 | Infiniti (Alcon), 2.75-mm incision, Healon, DAC, 2.75mm, Healon, longitudinal or torsional, one experienced operator  Noncontact specular microscopy | Incl.: Senile cataract with grade 2 or 3 nuclear brunescence scored with Oxford Clinical Cataract Classification and Grading System  Excl.: pseudoexfoliation syndrome or other severe ocular comorbidity (e.g. trauma); previous intraocular ocular surgery; corneal disease (dystrophy, corneal scarring); preoperative ECD <1500 cells/mm^2^ | 3 mo | 7.2% & 7.1%  NS |
| Cho Korea 2010a | Prospective study looking at ECL in consecutive eyes divided according to ACD (shhallow, intermediate, deep) | 94 | Machine not indicated, 3.0-mm incision, 1.2% sodium hyaluronate, Stop&Chop, one experienced operator  Noncontact specular microscopy | Excl.: History of previous ocular surgery or inflammation; glaucoma; corneal pathology; trauma; intraoperative complications (e.g. posterior capsule rupture); postoperative complications | 1 wk  6 wk  3 mo | 17.8%, 26.0%, 15.1%  15.6%, 25.4%, 17.7%  17.0%, 25.4%, 16.9%  NS |
| Cho Korea 2010b | Prospective study examining role of anterior segment parameters measured by Pentacam | 88 | Machine not indicated, 3.0-mm incision, 1.2% sodium hyaluronate, Stop&Chop, one experienced operator  Noncontact specular microscopy | Excl.: Significant corneal opacity; previous intraocular surgery; trauma; glaucoma, uveitis, Fuchs’ endothelial dystrophy, other abnormalities that could cause significant endothelial cell impairment independent of surgery; eyes with small pupils that required iris retractors, intraoperative complications such as posterior capsule rupture; postoperative complications. | 3 d  2 mo | 6.5%  16.8% |
| Baradaran Iran 2009 | RCT comparing high and low vacuum in high cataract grade | 30 high, 30 low | Whitestar Sovereign (Advanced Medical Optics), 2.8-mm incision, Coatel, Stop&Chop, one experienced operator  Noncontact specular microscopy | Incl.: 50 to 70 years 3+ nuclear sclerosis  Excl.: Previous corneal pathology (dystrophic or degenerative such as FECD or advanced trachoma); pseudoexfoliation syndrome; history of intraocular surgery; glaucoma, ocular hypertension, anterior uveitis; DM; ACD <2.5 mm or >4.0 mm; AL <21.0 mm or >25.0 mm; ECD <1500 cells/mm2; polymegathism (CV 0.4); keratometric astigmatism greater than 1.5 diopters; history of contact lens use,;intraoperative complications (posterior capsule rupture with or without vitreous loss); postoperative uveitis; postoperative surgical wound leakage; deep-set eye; dilated pupil <6.0 mm | 3 mo | 9.6% & 9.0%  NS |
| Lee Korea 2009 | RCT comparing 2 incision sizes (1.8 and 2.2 mm). The 2 groups were also divided into 3 subgroups based on LOCS III NO2–4 grade | 43 with 1.8, 43 with 2.2  20 & 21 in NO2  11 & 11 in NO3  12 & 12 in NO4 | Stellaris (Bausch&Lomb), 1.8/2.2-mm incision, Hyal Plus, DAC, one experienced surgeon  Noncontact specular microscopy | Incl.: LOCSIII grade II–IV nuclear or corticonuclear cataract  Excl.: Cataract hardness >NO5; ocular comorbidity; AL >25.0 mm; corneal dystrophy; corneal scarring; ECD <1500 cells/mm2; diabetes; use of oral steroidal agents; dermatological disease | 2 mo | NO2: 10% & 11.7%  NO3: 13.7% & 8.9%  NO4: 20.6% & 12.0% |
| Storr-Paulsen Denmark 2008 | RCT comparing phaco-chop and DAC | 60 eyes (30 & 30) | Millennium (Bausch&Lomb), 2.75-mm incision, Vitrax, DAC/phacochop, one experienced operator  Noncontact specular microscopy | Excl.: Corneal pathology; pseudoexfoliation; history of ocular trauma or intraocular surgery; intraocular inflammation; diabetes mellitus; <40 years; preoperative pupil dilation <4.0 mm; ECD <1500 cells/mm2; ACD <2.5 mm; surgical complications; preoperative eye medication | 3 mo  12 mo | 6.3% & 5.7%  5.0% & 3.5%  NS |
| Pereira Brazil 2006 | RCT comparing stop-chop and nuclear preslice ^a^ | 50 eyes with NO3NC3 or NO4NC4 | Legacy 20000 (Alcon), incision size not stated, Celoftal, Stop&Chop, one experienced operator  Specular microscopy | Incl.: LOCSIII grade NO3 NC3 and NO4 NC4 nuclear cataract  Excl.: Cornea guttata, FECD; pseudoexfoliation; glaucoma; dry eye; history of uveitis; previous eye surgery; diabetes mellitus; intraoperative and postoperative complications (e.g. posterior chamber rupture with vitreous loss, IOL decentration, Descemet’s membrane detachment, or exacerbated postoperative inflammation with ocular hypertension) | 3 mo | 8.7% both  NS |
| Lundberg Sweden 2005 | Prospective case series examining the relationship between CCT and ECL | 10 with <5% CCT increase, 10 with 6–20% increase, 10 with >21% increase | Legacy 20000 (Alcon), 3.0-mm incision, DuoVisc, DAC, two experienced operators  Noncontact specular microscopy | Incl.: 30 cases (aged 57–88 years) of uneventful phacoemulsification chosen for follow-up based on the increase in their corneal thickness within a 1-mm zone at the center of the cornea at the first postoperative day - first 10 patients with a <5% increase, the first 10 with 6–20% increase, first 10 with >20% increase | 1 d  1 mo  2 mo  3 mo | 2.2%  15.4%  18%  17.4% |
| O’Brien Ireland 2004 | Prospective case series looking for risk factors of ECL after phacoemulsification by a junior resident | 40 | Millenium (Bausch&Lomb), 2.75-mm incision, Viscoat, DAC, one resident with 100 phacoemulsification procedures under direct supervision of experienced operator  Noncontact specular microscopy | Excl.: Preexisting corneal pathology; ECD <1400 cells/mm2 | 3-4 w | 11.6% |
| Bourne UK 2004 | RCT comparing phacoemulsification with ECCE ^a^ | 223 phacoemulsification, 210 ECCE | Master 10000 (Alcon), 3.2-mm incision, Provisc, DAC, multiple experienced surgeons  Noncontact specular microscopy | Excl.: LOCIII grade 5 cataract; combined surgical procedures; other eye disorders capable of compromising vision (FECD, guttata >10 lesions in field illuminated by a Haag-Streit slit length of 6 mm and 10 marks on the width scale; corneal scarring apart from small nebulae); axial length >26.5 mm; <40 years | 12 mo | 10.5% & 9.1%  NS |
| Walkow Germany 2000 | Prospective case series identifying factors that relate to ECL | 50 | Machine not specified, 4.0-mm incision, soium hyaluronate, DAC, one experienced operator  Noncontact specular microscopy | Excl.: Astigmatism >2.5 D; significant corneal opacity; pseudoexfoliation syndrome; previous intraocular surgery; FECD; glaucoma; history ofanterior uveitis, dry-eye syndrome, iris neovascularization, or other abnormalities that could cause significant endothelial cell impairment independent of surgery | 12 mo | 8.5% |
| Hayashi Japan 1996 | Prospective case series examining risk factors for ECL | 859 | Premiere (Bausch&Lomb), 1% sodium hyaluronate, bimanual cracking procedure, one experienced operator  Noncontact specular microscopy | Excl.: History of previous ocular surgery or inflammation; abnormal findings by slitlamp biomicroscopic examination; pupil diameter >4.0 mm; ECD <1500 cells/mm2 | 3 mo | 4%, 8%, 10%, 12%, 15% with cataract grades 1–5 |
| Dick Germany 1996 | RCT comparing IOL implanted through 3.5-mm incision or 5.0-mm incision after phacoemulsification | 28 with 3.5, 30 with 5.0 | Premiere (Bausch&Lomb), Healon, DAC, one experienced operator  Contact specular microscopy  3.0mm, 1 exp, Healon, Storz Premiere, DAC & contact specular microscopy | Excl.: Corneal pathology; pseudoexfoliation syndrome | 12 mo | 6.7% & 7.3% |
| Zetterstrom Sweden 1995 | Case series study of ECL in phacoemulsification | 64 | Phaco Plus (Allergan Medical optics), 3.2-mm incision, Healon, DAC, one experienced operator  Specular microscopy | Excl.: Eye diseases other than cataracts (e.g. exfoliation syndrome); diabetes mellitus | 3 mo | 4.0% |
| Sugar USA | Case series  First study showing ECL after phacoemulsification | 70 compared to untreated contralateral eye | Specular microscopy |  | ≥1 mo | 33.8% |

^a^ In studies with a non-standard phacoemulsification (FLACS, ECCE, or nuclear preslice) or diabetic group, only univariate/multivariate analyses that related to the conventional phacoemulsification or non-diabetic control group were considered.

ACD, anterior chamber depth; AL, axial length; BCVA, best corrected visual acuity; BSS, balanced saline solution; CCT, central corneal thickness; CV, coeficient of variation; DAC, Divide-and-conquer; DM, diabetes mellitus; ECCE, extracapsular cataract surgery; ECD, endothelial cell density; ECL, endothelial cell loss; FECD, Fuchs’endothelial corneal dystrophy; FLACS, femtosecond laser-assisted cataract surgery; IOL, intraocular lens; IOP, intraocular pressure; LOCS, Lens Opacities Classification System; MICS, manual small-incision cataract surgery; NO, nuclear opacity; NSAID, non-sterioidal anti-inflammatory drug; OCT, optical coherence tomography; OVD, ophthalmic viscosurgical device; RCT, randomized controlled trial; SICS, small-incision cataract surgery.
