# Supplementary material for: Narrative review after post-hoc trial analysis of factors that predict corneal endothelial cell loss after phacoemulsification: Tips for improving cataract surgery research
Source: PLoS One. 2024 Mar 21;19(3):e0298795. doi: 10.1371/journal.pone.0298795 (PMC10956851; doi:10.1371/journal.pone.0298795)
Supplement: S2 Table — (DOCX) [file pone.0298795.s003.docx]

## Supplementary Table S2.

Summary of exclusion criteria used for the studies shown in Table 1 (where applicable, control group only)

| **Author date ref** | **Preop ECD** | **3-12 mo ECL** | **Exclusion criteria reported** | | | | | | |
| --- | --- | --- | --- | --- | --- | --- | --- | --- | --- |
|  |  |  | **Other corneal pathology, including high IOP** | **Previous eye surgery** | **Ocular trauma** | **Additional procedures** | **Preoperative ECD <1500, <2000, <1900, <1800, <2300** | **DM** | **Other** |
| Present study* | 2386±286 | 13 | X | X | X | X | X | X | VA<0.2 LogMAR |
| Mahmoud 2021* | 2799±350 | 6.4 | X | X |  |  | X | X |  |
| Budiman 2021* | 2420-2420 |  | X | X |  |  | X |  | Pseudoexfoliative syndrome; zonnular weakness; perioperative complications |
| Beato 2021* | 2421±304 | 20.7 | X |  |  |  | X | X |  |
| Joo 2021** | 2714±402 | 11.4 | X | X | X |  |  | X |  |
| Dzhaber 2021* | 2644±309 | 8.0 | X |  |  |  |  |  | Complicated pc |
| Choi 2019** | 2792±351 |  | X | X | X |  | X | X | Pc >10 years ago; poor pupillary dilatation; complicated pc |
| Krarup 2019* | 2673-2734 | 17 | X | X |  |  |  |  |  |
| Ganesan 2019* | 2337±363 | 18.1 | X |  |  |  |  |  | Age>40 |
| Perone 2018* | 2462±67 |  | X |  |  |  |  |  |  |
| Al-Osaily 2018* | 2550±336 | 17.3 | X |  |  |  |  |  |  |
| Singh 2017* | 2757±284 |  | X | X |  |  | X |  |  |
| Misra 2015* | 2384±438 | 9 | X | X | X |  |  | X | Contact lens wear; sutures required |
| Mehra 2015* | 2574 |  | X | X | X |  | X | X | Complicated pc |
| Atas 2014* | 2327-2392 |  | X | X | X |  | X |  | Age>50; poor pupillary dilation; complicated pc |
| Mayer 2014* | 2548±280 |  | X | X |  |  |  |  |  |
| Orski 2014* | 2445 | 13.1 |  |  |  |  |  |  |  |
| Conrad 2013* | 2452±258 | 13.7 | X |  |  |  |  |  | Poor pupillary dilation; NSAIDs in previous 3 mo; age<22 |
| Soliman 2012* | 2527±186 | 15.4 | X |  |  |  | X |  | Poor pupillary dilatation |
| Gonen 2012* | 2453-2599 |  | X | X |  |  | X | X | Age>50; poor pupillary dilatation |
| Takacs 2012* | 2841±215 | 10.5 | X |  |  |  |  |  |  |
| Faramarzi 2011* | 2544±299 | 13.6 | X | X | X |  | X | X | Poor pupillary dilatation; complicated pc; postoperative inflammation |
| Mathew 2011* | 1921±322 | 16.6 | X | X |  |  |  | X | Age-matched to DM group |
| Lucena 2011* | 2836±50 |  | X | X |  |  |  |  | Any condition that could impede specular microscopy; non-age-related cataract |
| Reuschel 2010* | 2365-2426 | 7.1 | X | X |  |  | X |  |  |
| Cho 2010a* | 2442-2725 | 16.9 | X | X | X |  |  |  | Complicated pc |
| Cho 2010b* | 2590±413 |  | X | X | X |  |  |  | Poor pupillary dilatation; complicated pc |
| Baradaran 2009* | 2541±305 | 9 | X | X |  |  | X | X | Age 50-70; poor pupillary dilatation; deepset eye; ACD<2.5mm or >4.0 mm; AL <21 mm or >25 mm; astigmatism >1.5D; contact lens use; complicated pc |
| Lee 2009** | 2532±332 |  | X |  |  |  | X | X | NSAID; AL >25 mm |
| Storr 2008* | 2742±424 | 3.5, 5.0 | X | X | X |  | X | X | Age<40; ACD<2.5; complicated pc |
| Pereira 2006** | 2560 | 8.7 | X | X |  |  |  | X | Complicated pc |
| Lundberg 2005* | 2752±681 | 17.4 |  |  |  |  |  |  | Senior surgeon cases |
| O’Brien 2004* | 2521±342 |  |  |  |  |  |  |  |  |
| Bourne 2004* | 2460±390 | 9.1 | X | X |  |  |  |  | Age<40; AL>26.5 mm |
| Walkow 2000* | 2397 | 8.5 | X |  |  |  |  |  | Astigmatism>2.5D |
| Hayashi 1996** | Not stated |  | X |  |  |  | X |  | Poor pupillary dilatation |
| Dick 1996* | 2352±261 | 7.3 | X |  |  |  |  |  |  |
| Zetterstrom 1995* | 3136±601 | 4.0 | X |  |  |  |  | X |  |

*Used one eye/patient for analyses.

**Sometimes used bilateral eyes for analyses.

ACD, anterior chamber depth; AL, axial length; DM, diabetes mellitus; ECD, endothelial cell density; ECL, endothelial cell loss; IOP, intraocular pressure; mo, month; NSAID, non-sterioidal anti-inflammatory drug; pc, phacoemulsification; VA, visual acuity; X, exclusion criteria reported.
