# Supplementary material for: Narrative review after post-hoc trial analysis of factors that predict corneal endothelial cell loss after phacoemulsification: Tips for improving cataract surgery research
Source: PLoS One. 2024 Mar 21;19(3):e0298795. doi: 10.1371/journal.pone.0298795 (PMC10956851; doi:10.1371/journal.pone.0298795)
Supplement: S3 Table — (DOCX) [file pone.0298795.s004.docx]

## Supplementary Table S3.

Comparison of eyes for which 2-hour CCT data were (n=234) and were not (n=41) available

| Variable | Eyes with 2-hour CCT  n=234 | Eyes without 2-hour CCT  n=41 | P* |
| --- | --- | --- | --- |
| Age | 74±9 | 74±11 | 0.79 |
| Sex | 132 (56) | 27 (66) | 0.31 |
| Cataract hardness, LOCSIII grade |  |  | 0.80 |
| NS1/2 | 41 (18) | 7 (17) |  |
| NS3 | 112 (48) | 22 (54) |  |
| NS4/5 | 81 (35) | 12 (29) |  |
| Preoperative CCT | 561±37 | 554±28 | 0.17 |
| Surgical technique | 127 (54) | 17 (41) | 0.17 |
| Intervention time, min:sec | 5.63±1.13 | 6.71±3.81 | 0.08 |
| EPT | 6.50±3.39 | 5.79±3.37 | 0.22 |
| 3-month ECL | 13%±15% | 13%±14% | 0.71 |

The data were expressed as mean±standard deviation or n (%), as appropriate.

* Student’s *t-*test or Fisher’s exact test.

ECL, endothelial cell loss; EPT, effective phaco time; LOCSIII, Lens Opacity Classification System III; NS, nuclear sclerosis.
