# Supplementary material for: Narrative review after post-hoc trial analysis of factors that predict corneal endothelial cell loss after phacoemulsification: Tips for improving cataract surgery research
Source: PLoS One. 2024 Mar 21;19(3):e0298795. doi: 10.1371/journal.pone.0298795 (PMC10956851; doi:10.1371/journal.pone.0298795)
Supplement: S4 Table — (DOCX) [file pone.0298795.s005.docx]

**Supplementary Table S4. Correlation coefficients for the relationship between cataract density and EPT in the literature**

| **Ref** | **Type sampling** | **N** | **Cataract density measuremt** | **Cataract grade** | | | | | | | | **Cataract density-EPT correlation** |
| --- | --- | --- | --- | --- | --- | --- | --- | --- | --- | --- | --- | --- |
|  |  |  |  | **1** | **2** | **3** | **4** | **5** | **6** | **Range** | **Mean** |  |
| Our study | Conv | 275 | LOCS | 1% | 16% | 49% | 33% | 1% | - | 1-5 | 3.2 | r=0.241 |
| Mehra 2015 [14] | Consec | 500 | LOCS | 19% | 34% | 26% | 21% | - | - | 1-4 | 2.5 | r=0.981 |
| Soliman 2012 [19] | Consec | 120 | LOCS | 11% | 19% | 33% | 20% | 18% | - | 1-5 | 3.1 | r=0.520 |
| Dzhaber 2020 [41] | Conv | 134 | LOCS | 9% | 25% | 61% | 5% | - | - | 1-4 | 2.6 | r=0.48 |
| Feng 2022 [53] | Conv | 381 | LOCS | - | - | - | - | - | - | 1-5 | 3.3 | r=0.582 |
| Faria 2018 [83] | Consec | 50 | LOCS | 20% | 30% | 30% | 20% | - | - | 1-4 | 3.8 | r=0.507 |
| Makhot 2018 [84] | Consec | 69 | LOCS | - | 32% | 32% | 35% | 4% | - | 2-5 | 3.6 | r=0.55 |
| Lim 2014 [85] | Consec | 70 | LOCS | - | - | - | - | - | - | 1-5 | 3.0 | r=0.753 |
| Gupta 2013 [86] | CD gps | 100 | LOCS | - | 25% | 25% | 25% | 25% | 25% | 3-6 | - | r=0.607 |
| Wang 2021 [87] | Consec | 1222 | LOCS | 8% | 19% | 28% | 19% | 17% | 9% | 1-6 | 3.9 | r=0.373 |
| Faria 2018 [83] | Consec | 50 | Pentacam | 20% | 30% | 30% | 20% | - | - | 1-4 | 3.8 | r=0.596 |
| Makhot 2018 [84] | Consec | 69 | Pentacam | - | 32% | 32% | 35% | 4% | - | 2-5 | 3.6 | r=0.40 |
| Lim 2014 [85]] | Consec | 70 | Pentacam | - | - | - | - | - | - | 1-5 | 3.0 | r=0.753 |
| Gupta 2013 [86] | CD gps | 100 | Pentacam | - | 25% | 25% | 25% | 25% | 25% | 3-6 | - | r=0.847 |
| Abell 2013 [88] | Consec | 51 | Pentacam | - | - | - | - | - | - | - | 2.5 | r=0.684 |
| Belikova 2013 [89] | Consec | 55 | Pentacam | 12% | 49% | 27% | 7% | 4% | - | - | 2.4 | r=0.531 |
| Patricio 2013 [90] | Tmt gps | 30 | Pentacam | 20% | 20% | 20% | 37% | 3% | - | 1-5 | 2.8 | r=0.924 |
| Wang 2021 [87] | Consec | 1222 | Other | 8% | 19% | 28% | 19% | 17% | 9% | 1-6 | 3.9 | r=0.427 |
| Mandelblum [91] | Random | 596 | Other | 10% | 41% | 40% | 10% | 1% | - | 1-5 | 2.6 | rho=0.8 |
| Mean |  |  |  |  |  |  |  |  |  |  | 3.2 | r=0.61 |

CD, cataract density; consec, consecutive; conv, convenience; gps, groups; Tmt, treatment.
