# Supplementary material for: Narrative review after post-hoc trial analysis of factors that predict corneal endothelial cell loss after phacoemulsification: Tips for improving cataract surgery research
Source: PLoS One. 2024 Mar 21;19(3):e0298795. doi: 10.1371/journal.pone.0298795 (PMC10956851; doi:10.1371/journal.pone.0298795)
Supplement: Attachment — Submitted filename: Response to Reviewers.docx [file pone.0298795.s006.docx]

**Point-by-point Response to Reviewer Comments**

# Editor Comments

Please include the following items when submitting your revised manuscript:

- A rebuttal letter that responds to each point raised by the academic editor and reviewer(s). You should upload this letter as a separate file labeled 'Response to Reviewers'.
- A marked-up copy of your manuscript that highlights changes made to the original version. You should upload this as a separate file labeled 'Revised Manuscript with Track Changes'.
- An unmarked version of your revised paper without tracked changes. You should upload this as a separate file labeled 'Manuscript'.

**Reply:** We have uploaded a rebuttal letter, a revised manuscript with Track Changes, and a revised manuscript without tracked changes.

Please ensure that your manuscript meets PLOS ONE's style requirements, including those for file naming. The PLOS ONE style templates can be found at 
<https://journals.plos.org/plosone/s/file?id=wjVg/PLOSOne_formatting_sample_main_body.pdf>

and 
<https://journals.plos.org/plosone/s/file?id=ba62/PLOSOne_formatting_sample_title_authors_affiliations.pdf>

**Reply:** We have changed the formatting of the paper and file names according to the instructions in the links.

2. Please ensure that you have specified a) Did participants provide their written or verbal informed consent to participate in this study?
b) If consent was verbal, please explain i) why written consent was not obtained, ii) how you documented participant consent, and iii) whether the ethics committees/IRB approved this consent procedure.

**Reply:** All subjects provided **written** informed consent before randomization. This was specified in the Study design and ethics section. *(Line 112)*

3. We note that you have indicated that data from this study are available upon request. PLOS only allows data to be available upon request if there are legal or ethical restrictions on sharing data publicly. For more information on unacceptable data access restrictions, please see http://journals.plos.org/plosone/s/data-availability#loc-unacceptable-data-access-restrictions.

In your revised cover letter, please address the following prompts:

a) If there are ethical or legal restrictions on sharing a de-identified data set, please explain them in detail (e.g., data contain potentially sensitive information, data are owned by a third-party organization, etc.) and who has imposed them (e.g., an ethics committee). Please also provide contact information for a data access committee, ethics committee, or other institutional body to which data requests may be sent.

b) If there are no restrictions, please upload the minimal anonymized data set necessary to replicate your study findings as either Supporting Information files or to a stable, public repository and provide us with the relevant URLs, DOIs, or accession numbers. For a list of acceptable repositories, please see <http://journals.plos.org/plosone/s/data-availability#loc-recommended-repositories>.

We will update your Data Availability statement on your behalf to reflect the information you provide.

**Reply:** The datasets generated during and/or analyzed during the current study are not publicly available according to French Law No. 2018-493 of June 20, 2018 on the protection of personal data (The General Data Protection Regulation (Regulation (EU) 2016/679) (GDPR: article 9) but are available from the Clinical Research Support Platform (Plateforme d’Appui à la Recherche Clinique [PARC]) of the Regional Central Hospital (CHR) of Metz-Thionville on reasonable request (email: projetrecherche@chrmetz-thionville.fr, tel: +33 3 87 17 98 82). All non-archived data is subject to daily backups while all archived data is subject to duplicate storage at two different sites. This data processing is compliant with a baseline reference methodology (MR-004) to which the CHR MetzThionville signed a compliance commitment on October 8, 2018.

4. Please amend your list of authors on the manuscript to ensure that each author is linked to an affiliation. Authors’ affiliations should reflect the institution where the work was done (if authors moved subsequently, you can also list the new affiliation stating “current affiliation:….” as necessary).

**Reply:** We removed the affiliation “^3^ LORIA, STID Department, IUT of Metz, Lorraine University, Metz, Grand Est, France” and indicated the affiliation for Dr. Zevering

5. Please include captions for your Supporting Information files at the end of your manuscript, and update any in-text citations to match accordingly. Please see our Supporting Information guidelines for more information: http://journals.plos.org/plosone/s/supporting-information.

**Reply:** We have provided the captions for Supplementary Tables S1–S4 at the end of the manuscript.

# Reviewer Comments

# Reviewer #1:

This manuscript seeks to identify predictive factors for endothelial cell loss after cataract surgery, a very important and clinically relevant topic. However, this topic has previously been extensively studied. This study does indeed have some strengths over other previously published manuscripts, with improvements to the methodology and study design. However this study does not change the overall conclusion and does not make any meaningful contribution to the literature, as older age and dense cataract are already well-known and widely accepted predictors of endothelial cell loss.

**Reply:** Thank you very much for taking the time to review and comment on our manuscript. We also appreciate your positive comments about our methodology and study design. We believe that our post-hoc trial data in the context of our narrative review add substantially to the field because:

- There is significant confusion in the field about the relationship between effective phaco time (EPT) and cataract density and how this affects endothelial-cell loss (ECL): multivariate analyses that included both EPT and cataract density showed that cataract density, not EPT, predicted ECL even though it is widely thought that the cataract density-ECL relationship is secondary to the effect of EPT on ECL. Our narrative review showed that this confusion likely reflects collinearity between cataract density and EPT and the inappropriate inclusion of these two variables in the same multivariate analysis.
- In unraveling this confusion, we noted that the correlation between cataract density and EPT varies markedly between cohorts: even in cohorts with a broad range of cataract densities, the cataract density:EPT correlation ranges from low (e.g. r=0.25 in our study) to very high in others (e.g. r=0.98). We were also struck by the finding of Lee et al. that a cataract density-EPT-ECL relationship only emerged when short-incisions were used rather than long-incisions. We were also interested in the study of Cho et al. that showed the cataract density-EPT-ECL relationship varied with ACD. These findings suggested to us that there may be patient and surgical conditions that could alter the cataract density-EPT relationship.
- These findings in turn suggest that it will be necessary to carefully dissect the cataract density-EPT-ECL relationship to find patient and surgical factors that reduce ECL in phacoemulsification. To our knowledge, these complex links between patient/surgical factors and ECL have never been comprehensively synthesized in the literature.
- There are also many studies that do not find that older age predict ECL: only four of seven previous multivariate analyses found that age predicted ECL. Our narrative review suggests that this may reflect, at least in part, endothelial fragility that is due to additional ECL risk factors such as diabetes. This highlights the importance of including endothelial fragility-related variables in multivariate analyses searching for patient/surgical factors that associate with increased ECL.

Thus, our study elucidates a very large, highly inconsistent, and complex field and points the way to improving research on factors that predict phacoemulsification-induced ECL. Since this analysis has significantly informed our own ongoing research in this field, we believe it will be useful to others as well.

Line 100: It does not make sense to say exclusion criteria included "preexisting cornea" and "intraocular pressure," as these are just parameters. Do the authors mean pre-existing corneal pathology and elevated intraocular pressure?

**Reply:** Yes, we used semi-colons to signify that exclusion criteria included preexisting cornea pathology, IOP pathology, and posterior-segment pathology. We understand that it is confusing to read and made it clearer as follows:

“Exclusion criteria were: white/brown cataracts; insulin-dependent diabetes or diabetic retinopathy; preexisting cornea pathology; intraocular-pressure (IOP) pathology; posterior-segment pathology; preoperative endothelial-cell density (ECD) >1500 cells/mm^2^; pregnancy;...” *(Lines 120–122)*

Line 101: Do the authors mean endothelial cell density LESS than 1500 cells/mm2? ">" should be changed to "<"

**Reply:** Thank you for picking this up. Yes, it should be <1500 cells/mm^2^. We made the change. *(Line 122)*

Line 113: How did the surgeon decide to use DAC vs subluxation?

**Reply:** The patients were randomized to either of the two treatment arms. We made this clearer as follows:

“Briefly, the cohort consisted of a convenience series of 292 adult patients who had a nuclear (NO1–NO4; NC1–NC4), cortical (C1–C5), or posterior subcapsular (P1–P5) cataract, as determined by using the Lens Opacities Classification System (LOCS)III classification [51], and a best spectacle-corrected visual acuity (BSCVA) of >+0.2 logMAR and were randomized to undergo either subluxation (*n*=148) or DAC (*n*=144).” *(Lines 115–120)*

Line 143: What were the technical reasons why surgical technique was altered?

**Reply:** To address this point, we added the following text to the Methods:

“The per-protocol trial data were analyzed: 15 of the 292 phacoemulsifications had been converted to the other method for technical reasons (an overly soft core, poor pharmacologically induced pupillary dilation, significant anterior chamber narrowness, and severely hard crystalline lens).” *(Lines 165–168)*

Lines 394-405: This discussion about hypothetical conditions needs to be removed or significantly altered. This discussion is meaningless because conditions A-E have not been defined. Moreover, hypothetical results have no place in a manuscript like this.

**Reply:** We agree and have deleted all of the texts and Fig. 2 about the hypothetical cataract grade-EPT relationships.

# Reviewer #2:

The investigators take care to note that this is a retrospective or post hoc look of a larger well designed cilnical trial with what appears to be an adequate sample size. The multivariate approach appears to be somewhat convincing in that only an older age (beta=0.2%, p=0.049) and higher EPT (beta=1.2%, p=0.0002) predicted 3-month ECL. Cataract density was significant on univariate (p=0.04) but not multivariate analysis. Study limitations are noted including the fact that they are currently conducting a large-scale prospective study assessing the impact of these and other variables on ECL, particularly in the context of different cataract grades.

All the statistical tools were in place for addressing this post hoc concern and clearly the investigators demonstrated that sufficient leads are in place to confirm these results. Apparently available data was collected as seen in Tables 1 and 2 to investigate the preoperative, operative and demographic data. Univariate and multivariate results are demonstrated clearly in tables 3 and 4.

**Reply:** Thank you very much for taking the time to read and comment on our manuscript, and for your positive comments.

It appears that the three month time is chosen for investigating the ECL. One wonders if that is the only time point considered in this analysis or other time points could have been considered to investigate a time dependent influence of the variables on the ECL. This is a non clinical statistical reviewer wondering such.

**Reply:** Yes, it is a good point that the relationships between the variables could potentially change over time. However, we had to choose the 3-month point because our 12-month data were complicated by a large loss-to follow-up (29%). By contrast, only 6% were lost to follow-up at 3 months. To address this point, we added the following text to the Methods:

“ECD measured 3 months after surgery was used to calculate ECL at 3 months relative to baseline. The 3-month timepoint was chosen for analysis rather than the 12-month timepoint in our trial because loss to follow-up was 6% at 3 months and 29% at 12 months.” *(Lines 151–154)*

# Reviewer #3:

Thank you for the opportunity to review this paper by Perone and colleagues, In general, it is a very well written paper about an important but also complex topic, that of the effects of phacoemulsification on endothelial cell loss.

I believe the methods, analysis and the results of the paper are valid, and in line with the current literature that the authors have extensively reviewed.

**Reply:** Thank you very much for taking the time to review and comment on our paper, and for your positive comments.

My main concern is directed towards the lengthy discussion in the paper, as well as some of the suggestions made on future research. As a clinical research paper, I would say that the discussion is far too long and veers into the territory of being a review paper on its own. The authors may wish to consider discussing their own personal results, and conjecture on why they do or do not affect 3 month ECL, without referencing all of past research that has been done. The actual review of the literature is very comprehensive, but may be better served as a separate paper in another journal.

**Reply:** Indeed, our manuscript describes a posthoc trial analysis that is interpreted in the context of a very large, complex, and confusing field in the form of a narrative review. We decided to take this approach because although our multivariate analyses did not yield particularly novel predictive factors for endothelial-cell loss (ECL), our comprehensive review and analysis of the literature revealed important aspects about the cataract density-EPT-ECL relationship that should be taken into account when searching for patient/surgical factors that influence ECL after phacoemulsification. We recognize that our approach – a narrative review centered on our posthoc analysis – is somewhat unorthodox but feel that it will help illustrate how to improve research in this field. Indeed, this analysis has significantly informed our own ongoing research in this field. Therefore, we believe it will be useful to others as well.

To address this point, we changed the title as follows:

“Narrative review after posthoc trial analysis of factors that predict corneal endothelial cell loss after phacoemulsification: tips for improving cataract surgery research” *(Lines 3–6)*

One of the suggestions made by the authors in improving the research in this field is to clearly define and document the cataract density of the study cohort. They state that this is important as “Our analysis shows that cataract density can greatly shape the ECL-inducing effects of EPT/CDE”. While I believe that is indeed important, the results of this study do not directly demonstrate that association between cataract density and ECL on multivariate analysis. Perhaps they drew this conclusion based on the 5 previous metanalyses that did show a correlation (lines 284-285)?

**Reply:** It is a widely held view in the field that cataract density shapes ECL because more phaco power (EPT) is needed to process a harder lens. This view is borne out by the literature (see new Supplementary Table S4 below): the average correlation coefficient for the relationship between cataract grade and EPT is r=0.61. This correlation was also significant in our study on univariate analysis (r=0.25; p<0.0001).

New Supplementary Table S4. Correlation coefficients for the relationship between cataract density and EPT in the literature

| Ref | Type sampling | N | Cataract density measuremt | Cataract grade | | | | | | | | Cataract density-EPT correlation |
| --- | --- | --- | --- | --- | --- | --- | --- | --- | --- | --- | --- | --- |
|  |  |  |  | 1 | 2 | 3 | 4 | 5 | 6 | Range | Mean |  |
| Our study | Conv | 275 | LOCS | 1% | 16% | 49% | 33% | 1% | - | 1-5 | 3.2 | r=0.241 |
| Mehra 2015 [14] | Consec | 500 | LOCS | 19% | 34% | 26% | 21% | - | - | 1-4 | 2.5 | r=0.981 |
| Soliman 2012 [19] | Consec | 120 | LOCS | 11% | 19% | 33% | 20% | 18% | - | 1-5 | 3.1 | r=0.520 |
| Dzhaber 2020 [41] | Conv | 134 | LOCS | 9% | 25% | 61% | 5% | - | - | 1-4 | 2.6 | r=0.48 |
| Feng 2022 [53] | Conv | 381 | LOCS | - | - | - | - | - | - | 1-5 | 3.3 | r=0.582 |
| Faria 2018 [83] | Consec | 50 | LOCS | 20% | 30% | 30% | 20% | - | - | 1-4 | 3.8 | r=0.507 |
| Makhoti 2018 [84] | Consec | 69 | LOCS | - | 32% | 32% | 35% | 4% | - | 2-5 | 3.6 | r=0.55 |
| Lim 2014 [85] | Consec | 70 | LOCS | - | - | - | - | - | - | 1-5 | 3.0 | r=0.753 |
| Gupta 2013 [86] | CD gps | 100 | LOCS | - | 25% | 25% | 25% | 25% | 25% | 3-6 | - | r=0.607 |
| Wang 2021 [87] | Consec | 1222 | LOCS | 8% | 19% | 28% | 19% | 17% | 9% | 1-6 | 3.9 | r=0.373 |
| Faria 2018 [83] | Consec | 50 | Pentacam | 20% | 30% | 30% | 20% | - | - | 1-4 | 3.8 | r=0.596 |
| Makhoti 2018 [84] | Consec | 69 | Pentacam | - | 32% | 32% | 35% | 4% | - | 2-5 | 3.6 | r=0.40 |
| Lim 2014 [85]] | Consec | 70 | Pentacam | - | - | - | - | - | - | 1-5 | 3.0 | r=0.753 |
| Gupta 2013 [86] | CD gps | 100 | Pentacam | - | 25% | 25% | 25% | 25% | 25% | 3-6 | - | r=0.847 |
| Abell 2013 [88] | Consec | 51 | Pentacam | - | - | - | - | - | - | - | 2.5 | r=0.684 |
| Belikova 2013 [89] | Consec | 55 | Pentacam | 12% | 49% | 27% | 7% | 4% | - | - | 2.4 | r=0.531 |
| Patricio 2013 [90] | Ttmt gps | 30 | Pentacam | 20% | 20% | 20% | 37% | 3% | - | 1-5 | 2.8 | r=0.924 |
| Wang 2021 [87] | Consec | 1222 | Other | 8% | 19% | 28% | 19% | 17% | 9% | 1-6 | 3.9 | r=0.427 |
| Mandelblum [91] | Random | 596 | Other | 10% | 41% | 40% | 10% | 1% | - | 1-5 | 2.6 | rho=0.8 |
| Mean |  |  |  |  |  |  |  |  |  |  | 3.2 | r=0.61 |

CD, cataract density; consec, consecutive; conv, convenience; gps, groups; Ttmt, treatment.

What is also notable in the table above is the wide range of correlation coefficients: they run from 0.25 in our study to 0.98 (see new Supplementary Fig S1 below). These disparities do not reflect different cataract grade distributions since most studies included a wide spread of grades.

New Supplementary Fig S1. Plot of correlation coefficients in the literature

|  | Mean cataract grade |
| --- | --- |
| 1 | 0.25 |
| 2 | 0.981 |
| 3 | 0.52 |
| 4 | 0.48 |
| 5 | 0.582 |
| 6 | 0.507 |
| 7 | 0.55 |
| 8 | 0.753 |
| 9 | 0.607 |
| 10 | 0.373 |
| 11 | 0.596 |
| 12 | 0.4 |
| 13 | 0.753 |
| 14 | 0.847 |
| 15 | 0.684 |
| 16 | 0.531 |
| 17 | 0.924 |
| 18 | 0.427 |

This variation in cataract grade-EPT relationship strength caught our eye when we read the RCT of Lee et al. that compared long and short corneal incisions (ref 29): they observed a cataract density-EPT-ECL relationship with short incisions but not long incisions (see shots of the Lee et al. figures below).

Thus, these data together with other studies detailed in the manuscript led to three conclusions: (1) “cataract density can greatly shape the ECL-inducing effects of EPT/CDE” **but** (2) this effect can be modified by surgical and patient (e.g. ACD) factors; **and** (3) therefore, to identify modifiable factors that shape ECL, it is essential to consider cataract density, which is often barely (or not) detailed in the literature (e.g. Feng et al. 2022, Lim et al. 2014, and Abell et al. 2013 in new Supplementary Table S4).

To address this important point, we added new Supplementary Table S4 and new Supplementary Fig S1 to the manuscript as well as the underlined Discussion text below:

“This cataract density-ECL relationship is widely thought to be secondary to the effect of EPT/CDE on ECD: more mechanical energy is needed to fragment harder cataracts. Indeed, in our study, cataract density correlated significantly with EPT on univariate analysis (r=0.25, p<0.0001) and the univariate association between ECL and cataract hardness disappeared on multivariate analysis.” *(Lines 332–336)*

Even so, even if there were an association between cataract density and EPT, the overall effect may yet be small, as their study results only found that ECL increased 1.2% for every second longer EPT (line 210).

We agree that EPT had a relatively small effect in our study. It should be noted that while several multivariate studies in the literature do find that EPT predicts ECL, some do not (see Table 1 of our manuscript). Moreover, one multivariate study (Ref 35) also shows a small effect of EPT whereas another shows a large effect (Ref 16). This variability likely reflects (i) variable cohort characteristics, (ii) the covariates that were included in the multivariate analysis, and (iii) in some cases, the erroneous inclusion of cataract density in multivariate analysis when it is collinear with EPT.

To address this point, we added the following text to the Discussion:

“However, the research on ECL has several important limitations that should be addressed to allow further improvements in phacoemulsification outcomes. One is that many studies use EPT/CDE as a surrogate of ECL. This is inappropriate for two reasons: (i) there are many RCTs where an intervention significantly affects EPT/CDE but not ECL [8,110,111] and vice versa [30,112,113]. (ii) EPT accounted for only 5.1% of the total ECL variation in our cohort. Baradaran et al. also observed that it was a weak predictor [35]. Notably, however, another study found CDE had a large effect [16]; this disparity is likely due to the covariates that were included and different cohort characteristics and consolidates the crucial importance of including other patient/surgical factors.” *(Lines 434–442)*

In terms of the limitation of the study, it would be prudent to address the fact that this study was a posthocc analysis of a trial that was primarily looking at the differences between two surgical techniques. As such, the sample size of this study, while large, may not have been sufficiently powered to identify variables that contribute to ECL loss. An example would again be cataract density, where there were very few cases with very soft (NS1+1) and very dense (NS5+) due to the convenience sampling. Finally, this is essentially the results of a single, very experienced surgeon, which may again result in systematic biases in the results.

**Reply:** These are excellent points. We have added the following text to the Limitations section:

“However, 82% of the cataracts in our study had NS3–4 density. Thus, the low frequencies of NS1, NS2, and NS5 cataracts may have precluded us from detecting variables that contribute significantly to ECL in such cases. Moreover, all surgeries were conducted by a single experienced surgeon: thus, our data may not be generalizable to other settings.” *(Lines 478–482)*
