# Supplementary material for: Narrative review after post-hoc trial analysis of factors that predict corneal endothelial cell loss after phacoemulsification: Tips for improving cataract surgery research
Source: PLoS One. 2024 Mar 21;19(3):e0298795. doi: 10.1371/journal.pone.0298795 (PMC10956851; doi:10.1371/journal.pone.0298795)
Supplement: Attachment — Submitted filename: Response to Reviewers.docx [file pone.0298795.s007.docx]

**Response to Reviewers**

# Editor comments

# Comment 1:

-Please replace the subsection title "Statistical analyses" with the title "Statistical analysis"

**Reply:** We have changed the subsection title to “Statistical analysis”.

## Comment 2:

-Please add a power analysis to determine if the sample size is adequate.

**Reply:** Before the PERCEPOLIS trial was conducted, a sample size calculation was conducted. This showed that to detect the expected endothelial-cell loss of 5–12% at 1 month (based on the literature at the time), 294 patients had to be recruited. In total, 292 were recruited. This sample size calculation was reported by our published article describing the primary endpoint of the trial (Perone et al. Corneal Endothelial Cell Loss After Endocapsular and Supracapsular Phacoemulsification. 2021. Cornea. 41;714).

However, we believe that the question being asked was, “Was the sample size sufficient for the multivariate analysis in the present study?” To address this, we added the following text and reference 57 to the Statistics section:

“*Multiple linear regression analysis was conducted to identify which of nine variables can independently predict ECL at 3 months. Given the cohort sample size (n=275) and the 1 in 10 rule of thumb in multivariate analysis that states one candidate predictor can be studied for every 10 patients,[57] the sample size was ample for this analysis*.” Lines 176–180

# Comment 3:

Please review your reference list to ensure that it is complete and correct.

**Reply:** The reference list has been reviewed.

# Reviewer Comments

Reviewer 2: My concern was with the adequacy of the follow up time. The authors apparently believe that the 3 month time is sufficient. If such is the case then the analysis will follow.

**Reply:** We chose to focus on the 3-month timepoint because there was a large loss to follow-up at the later timepoint in the trial (12 months; 93 of 292 lost = 32% loss). Thus, the 12-month cohort may have been selected in some way, meaning they may not have been fully representative of the general phacoemulsification patients. In addition, longitudinal studies show that post-phacoemulsification ECL has stabilized by 3 months: for example, Lass et al. (Am J Ophthalmol. 2019 208;211) showed that ECL was 8.3% and 8.7% at 3 and 24 months, respectively.

To address this point, we added the following text to the Methods section:

“*Although ECD and other data were collected from the PERCEPOLIS cohort at 1, 3, and 12 months, we chose the 3-month data for the present study because there was a large loss to follow-up at 12 months (32%), meaning there may have been some selection of the 12-month patients. Moreover, ECL is generally considered to have stabilized at 3 months [12,16,18,33,52]*.” (lines 125–128)
